# Supplementary material for: A Clinical Validation of a Diagnostic Test for Esophageal Adenocarcinoma Based on a Novel Serum Glycoprotein Biomarker Panel: PromarkerEso
Source: Proteomes. 2025 Jun 4;13(2):23. doi: 10.3390/proteomes13020023 (PMC12196998; doi:10.3390/proteomes13020023)

SUPPLEMENTARIES

Supplementary Table S1: MRM-MS peptide candidates

Precursor Ion	Product Ion	Uniprot Protein Code, Peptide seq., ion	CE	RT(min)
308.5151	218.1499	P00738.ILGGHLDAK.+3y2	12.8	5.36
308.5151	349.1850	P00738.ILGGHLDAK.+3y7+2	12.8	5.36
308.5151	109.5786	P00738.ILGGHLDAK.+3y2+2	12.8	5.36
490.7511	881.4264	P00738.VGYVSGWGR.+2y8	23.0	6.10
490.7511	661.3416	P00738.VGYVSGWGR.+2y6	23.0	6.10
490.7511	562.2732	P00738.VGYVSGWGR.+2y5	23.0	6.10
460.7349	389.1819	P00738.GSFPWQAK.+2b4	21.6	6.38
460.7349	776.4090	P00738.GSFPWQAK.+2y6	21.6	6.38
460.7349	629.3406	P00738.GSFPWQAK.+2y5	21.6	6.38
602.3220	1003.5207	P00738.VTSIQDWVQK.+2y8	28.5	6.33
602.3220	803.4046	P00738.VTSIQDWVQK.+2y6	28.5	6.33
602.3220	675.3461	P00738.VTSIQDWVQK.+2y5	28.5	6.33
508.3109	187.1077	P01009.SVLGQLGITK.+2b2	23.9	6.54
508.3109	829.5142	P01009.SVLGQLGITK.+2y8	23.9	6.54
508.3109	716.4301	P01009.SVLGQLGITK.+2y7	23.9	6.54
531.2975	819.4611	P01011.EIGELYLPK.+2y7	25.0	6.50
531.2975	633.3970	P01011.EIGELYLPK.+2y5	25.0	6.50
531.2975	520.3130	P01011.EIGELYLPK.+2y4	25.0	6.50
954.4835	399.2238	P01011.AVLDVFEEGTEASAATAVK.+2b4	45.8	6.81
954.4835	498.2922	P01011.AVLDVFEEGTEASAATAVK.+2b5	45.8	6.81
954.4835	1005.5211	P01011.AVLDVFEEGTEASAATAVK.+2y11	45.8	6.81
608.3690	888.5149	P01011.ITLLSALVETR.+2y8	28.8	7.24
608.3690	775.4308	P01011.ITLLSALVETR.+2y7	28.8	7.24
608.3690	688.3988	P01011.ITLLSALVETR.+2y6	28.8	7.24
547.8195	867.5047	P01011.NLAVSQVVHK.+2y8	25.8	5.39
547.8195	796.4676	P01011.NLAVSQVVHK.+2y7	25.8	5.39
547.8195	697.3991	P01011.NLAVSQVVHK.+2y6	25.8	5.39
621.8765	1042.6295	P02748.LSPIYNLVPVK.+2y9	29.5	6.81
621.8765	832.4927	P02748.LSPIYNLVPVK.+2y7	29.5	6.81
621.8765	343.2340	P02748.LSPIYNLVPVK.+2y3	29.5	6.81
621.8765	521.8184	P02748.LSPIYNLVPVK.+2y9+2	29.5	6.81
728.3594	1142.5477	P02748.AIEDYINEFSVR.+2y9	34.7	7.00
728.3594	864.4574	P02748.AIEDYINEFSVR.+2y7	34.7	7.00
728.3594	751.3733	P02748.AIEDYINEFSVR.+2y6	34.7	7.00
526.2876	653.3518	P02748.RPWNVASLIYETK.+3b5	23.3	6.70
526.2876	653.3505	P02748.RPWNVASLIYETK.+3y5	23.3	6.70
526.2876	540.2664	P02748.RPWNVASLIYETK.+3y4	23.3	6.70
610.8066	959.4945	P02790.NFPSPVDAAFR.+2y9	28.9	6.56
610.8066	862.4417	P02790.NFPSPVDAAFR.+2y8	28.9	6.56
610.8066	775.4097	P02790.NFPSPVDAAFR.+2y7	28.9	6.56
748.3430	1009.5214	P02790.YYC[CAM]FQGNQFLR.+2y8	35.7	6.92

748.3430	862.4530	P02790.YYC[CAM]FQGNQFLR.+2y7	35.7	6.92
487.2789	300.1707	P02790.LWWLDLK.+2b2	22.9	7.52
487.2789	674.3872	P02790.LWWLDLK.+2y5	22.9	7.52
487.2789	260.1969	P02790.LWWLDLK.+2y2	22.9	7.52
459.2506	715.4349	P03952.DSVTGTLPK.+2y7	21.5	5.51
459.2506	616.3665	P03952.DSVTGTLPK.+2y6	21.5	5.51
459.2506	515.3188	P03952.DSVTGTLPK.+2y5	21.5	5.51
514.7904	814.4417	P05546.TLEAQLTPR.+2y7	24.2	5.82
514.7904	685.3991	P05546.TLEAQLTPR.+2y6	24.2	5.82
514.7904	486.3035	P05546.TLEAQLTPR.+2y4	24.2	5.82
393.2476	228.1343	P05546.LNILNAK.+2b2	18.3	5.96
393.2476	558.3610	P05546.LNILNAK.+2y5	18.3	5.96
393.2476	445.2769	P05546.LNILNAK.+2y4	18.3	5.96
410.2524	185.1285	P05546.IAIDLFK.+2b2	19.1	6.81
410.2524	706.4134	P05546.IAIDLFK.+2y6	19.1	6.81
410.2524	635.3763	P05546.IAIDLFK.+2y5	19.1	6.81
660.3513	1007.5156	P06396.AGALNSNDAFVLK.+2y9	31.4	6.33
660.3513	893.4727	P06396.AGALNSNDAFVLK.+2y8	31.4	6.33
660.3513	200.1030	P06396.AGALNSNDAFVLK.+2b3	31.4	6.33
378.2367	585.3606	P06396.AVEVLPK.+2y5	17.5	5.55
378.2367	456.3180	P06396.AVEVLPK.+2y4	17.5	5.55
378.2367	244.1656	P06396.AVEVLPK.+2y2	17.5	5.55
915.4858	1187.6307	P06396.QTQVSVLPEGGETPLFK.+2y11	43.9	6.67
915.4858	1074.5466	P06396.QTQVSVLPEGGETPLFK.+2y10	43.9	6.67
346.7101	579.3289	P06396.IFVWK.+2y4	16.0	6.51
346.7101	432.2605	P06396.IFVWK.+2y3	16.0	6.51
346.7101	333.1921	P06396.IFVWK.+2y2	16.0	6.51
592.8297	943.5095	P27169.IQNILTEEPK.+2y8	28.0	6.04
592.8297	716.3825	P27169.IQNILTEEPK.+2y6	28.0	6.04
592.8297	603.2984	P27169.IQNILTEEPK.+2y5	28.0	6.04
942.4623	261.1598	P27169.IFFYDSENPPASEVLR.+2b2	45.2	6.88
942.4623	1016.4360	P27169.IFFYDSENPPASEVLR.+2b8	45.2	6.88
942.4623	868.4887	P27169.IFFYDSENPPASEVLR.+2y8	45.2	6.88
844.4236	1121.5334	P01012.GGLEPINFQTAADQAR.+2y10	40.4	6.44
844.4236	860.4221	P01012.GGLEPINFQTAADQAR.+2y8	40.4	6.44
844.4236	666.3388	P01012.GGLEPINFQTAADQAR.+2y12+2	40.4	6.44

Supplemental Table S2 – MRM reproducibility

Peptide sequence	Assay 01 (n=8)	Assay 02 (n=8)	Assay 03 (n=8)	Assay 04 (n=8)	Assay 05 (n=8)	Assay 06 (n=8)	Mean (n=6)	SD (n=6)	Inter-assay CV% (n=6)
ILGGHLDAK	10.78	10.40	10.48	9.92	9.24	7.93	9.79	1.060	11%
VGYVSGWGR	16.55	15.22	12.86	11.16	13.50	11.10	13.40	2.182	16%
GSFPWQAK	15.38	14.01	12.00	11.00	12.10	9.41	12.32	2.125	17%
VTSIQDWVQK	10.82	11.25	10.37	9.60	10.80	9.28	10.35	0.768	7%
SVLGQLGITK	4.15	4.76	3.34	3.03	3.69	3.08	3.68	0.675	18%
EIGELYLPK	3.60	3.39	3.14	2.71	3.01	2.79	3.11	0.343	11%
AVLDVFEEGTEASAATAVK	0.23	0.31	0.28	0.23	0.29	0.33	0.28	0.040	14%
ITLLSALVETR	2.00	2.55	2.10	1.97	2.45	2.64	2.28	0.295	13%
NLAVSQVVHK	0.75	0.77	0.83	0.86	0.82	0.74	0.79	0.050	6%
LSPIYNLVPVK	0.90	1.08	0.85	0.73	0.81	0.80	0.86	0.122	14%
AIEDYINEFSVR	0.21	0.24	0.19	0.18	0.16	0.19	0.19	0.029	15%
RPWNVASLIYETK	0.01	0.02	0.02	0.01	0.01	0.02	0.02	0.004	25%
NFPSPVDAAFR	5.83	7.12	5.31	4.88	5.14	6.52	5.80	0.871	15%
YYC[+57]FQGNQFLR	0.12	0.14	0.08	0.09	0.19	0.16	0.13	0.040	30%
LWWLDLK	1.26	1.41	0.58	0.76	0.78	0.61	0.90	0.347	39%
DSVTGTLPK	0.09	0.10	0.08	0.07	0.07	0.11	0.09	0.017	19%
TLEAQLTPR	1.09	1.10	1.01	0.98	0.91	0.79	0.98	0.118	12%
LNILNAK	0.45	0.45	0.44	0.44	0.41	0.44	0.44	0.016	4%
IAIDLFK	1.21	1.32	1.20	1.09	1.09	1.10	1.17	0.093	8%
AGALNSNDAFVLK	0.70	0.67	0.64	0.63	0.57	0.47	0.61	0.084	14%
AVEVLPK	0.80	0.80	0.76	0.68	0.66	0.52	0.70	0.108	15%
QTQVSVLPEGGETPLFK	0.21	0.20	0.20	0.20	0.18	0.15	0.19	0.023	12%
IQNILTEEPK	0.49	0.54	0.43	0.44	0.40	0.52	0.47	0.056	12%
IFVWK	0.53	0.48	0.33	0.35	0.36	0.22	0.38	0.111	29%
IFFYDSENPPASEVLR	0.26	0.30	0.29	0.27	0.28	0.25	0.28	0.018	7%

The values for each assay and each peptide are the peak area ratios of the peptide peak area to the ovalbumin peptide peak area.

MRM-LCMS method details

For MRM-LCMS analysis 100 µL of the digestion mixture was transferred to a 96-well plate for injection on an Ultimate 3000 HPLC system (Thermofisher). The LC parameters were as follows.

Supplementary Table S3 – LC Parameters

**Column**	Acclaim™ PepMap™ 100 C18 (2um particle size, 0.3mm diameter x 150mm length)			
**Mobile phase A**	0.1% FA in 2% MS grade ACN			
**Mobile phase B**	0.1% FA in 98% MS grade ACN			
**Injection Volume**	20 µL			
**Autosampler Temperature**	4 °C			
**Run Time**	9.5 minutes			
**Flow Rate**	10 µL/min			
**Gradient Profile**	Time	Module	Event	%B
	0	Pumps	Pump B conc.	10
	1	Pumps	Pump B conc.	10
	4	Pumps	Pump B conc.	20
	5	Pumps	Pump B conc.	25
	7	Pumps	Pump B conc.	40
	8	Pumps	Pump B conc.	98
	8.5	Pumps	Pump B conc.	98
	8.6	Pumps	Pump B conc.	10
	9.5	Pumps	Pump B conc.	10

The Sciex 6500+ mass spectrometer settings were as follows.

Supplemental Table S4 – MS Parameters

**Curtain gas**	35
**Collision gas**	Medium
**Ion Spray Voltage**	5000 V
**Interface Temperature**	150 °C
**GS1**	20
**GS2**	15
**Q1 resolution**	Unit
**Q3 resolution**	Unit

Skyline (version 24.1.0.199) was used for inspecting and processing the MRM data. The peak picking algorithm in Skyline was optimized for improved peak integration. The peak area for each peptide (the sum of all the transitions) was exported. The concentration of each peptide in each sample was presented as the peak area ratio of the native peptide area over the ovalbumin internal standard peak area.

Supplementary Table S5: Bivariate and cross-cohort analyses of all candidate peptides: Investigation of 25 peptide relative concentrations across PROBE-NET, Ochsner and VCB cohorts. Relative values of peptides were natural logarithm-transformed and median ratios between EAC and controls were compared across cohorts by Mann–Whitney U test and Kruskal–Wallis test. Significance denoted by p-value of >0.05 = n.s., <0.05 = *, <0.01=**, <0.001 = ***, <0.0001 = ****.

			EAC vs Controls (Negative or General) Mann–Whitney U P value			Kruskal–Wallis P value
**PROTEIN**	**UniProt ID**	**PEPTIDE SEQUENCE**	**PROBE-NET**	**Ochsner**	**VCB**	
**Haptoglobin**	P00738	ILGGHLDAK	0.0130 *	0.5458 n.s	1.96E-05 ****	3.64E-44****
		VGYVSGWGR	0.0266 *	0.0089 **	5.61E-07 ****	1.78E-42****
		GSFPWQAK	0.0216 *	0.0306 *	5.32E-07 ****	9.48E-28****
		VTSIQDWVQK	0.0162 *	0.0107 *	0.0004 ***	2.19E-38****
**Alpha-1-antitrypsin**	P01009	SVLGQLGITK	0.0006 ***	0.0004 ***	2.42E-07 ****	1.34E-26****
**Alpha-1-antichymotrypsin**	P01011	EIGELYLPK	0.0684 n.s	0.0031 **	7.65E-09 ****	2.82E-26****
		AVLDVFEEGTEASAATAVK	0.0803 n.s	0.0031 **	5.75E-05 ****	2.75E-45****
		ITLLSALVETR	0.0851 n.s	0.0048 **	0.0503 n.s	1.39E-30****
		NLAVSQVVHK	0.3509 n.s	0.1375 n.s	0.3700 n.s	1.57E-05****
**Complement C9**	P02748	LSPIYNLVPVK	0.0032 **	0.0003 ***	2.34E-06 ****	1.37E-21****
		AIEDYINEFSVR	0.0030 **	0.0003 ***	1.23E-05 ****	4.17E-28****
		RPWNVASLIYETK	0.0046 **	0.0019 **	2.25E-07 ****	0.000299***
**Hemopexin**	P02790	NFPSPVDAAFR	0.0913 n.s	0.0130 *	9.23E-05 ****	1.49E-40****
		YYC[+57]FQGNQFLR	0.0780 n.s	0.1721 n.s	0.0160 *	1.25E-43****
		LWWLDLK	0.0316 *	0.0089 **	2.21E-05 ****	1.48E-08****
**Plasma kallikrein**	P03952	DSVTGTLPK	0.0017 *	0.6665 n.s	0.2318 n.s	4.40E-39****
**Heparin cofactor 2**	P05546	TLEAQLTPR	0.1020 n.s	0.0841 n.s	0.0515 n.s	2.49E-47****
		LNILNAK	0.3046 n.s	0.2591 n.s	0.0007 ***	1.38E-36****
		IAIDLFK	0.2198 n.s	0.1223 n.s	2.02E-05 ****	7.36E-09****
**Gelsolin**	P06396	AGALNSNDAFVLK	0.0792 n.s	0.5845 n.s	0.2746 n.s	3.38E-47****
		AVEVLPK	0.0332 *	0.1375 n.s	0.0313 *	2.48E-47****
		QTQVSVLPEGGETPLFK	0.0792 n.s	0.3407 n.s	0.1732 n.s	2.50E-47****
		IFVWK	0.0244 *	1.0000 n.s	4.14E-05 ****	6.82E-46****
**Serum paraoxonase /arylesterase**	P27169	IQNILTEEPK	0.0067 **	0.3119 n.s	0.2731 n.s	5.54E-47****
		IFFYDSENPPASEVLR	0.0061 **	0.4031 n.s	0.4354 n.s	7.57E-16****

Supplementary Figure S1: Glycoprotein Multicollinearity: Assessment of inter- and intra- glycoprotein multicollinearity in PROBE-NET cohort. Multicollinearity was investigated using Kendall's tau to determine independence of peptides as covariates in model building and visually assessed by bubble-heat map correlation matrix for positive and negative intra- and inter-protein relationships.

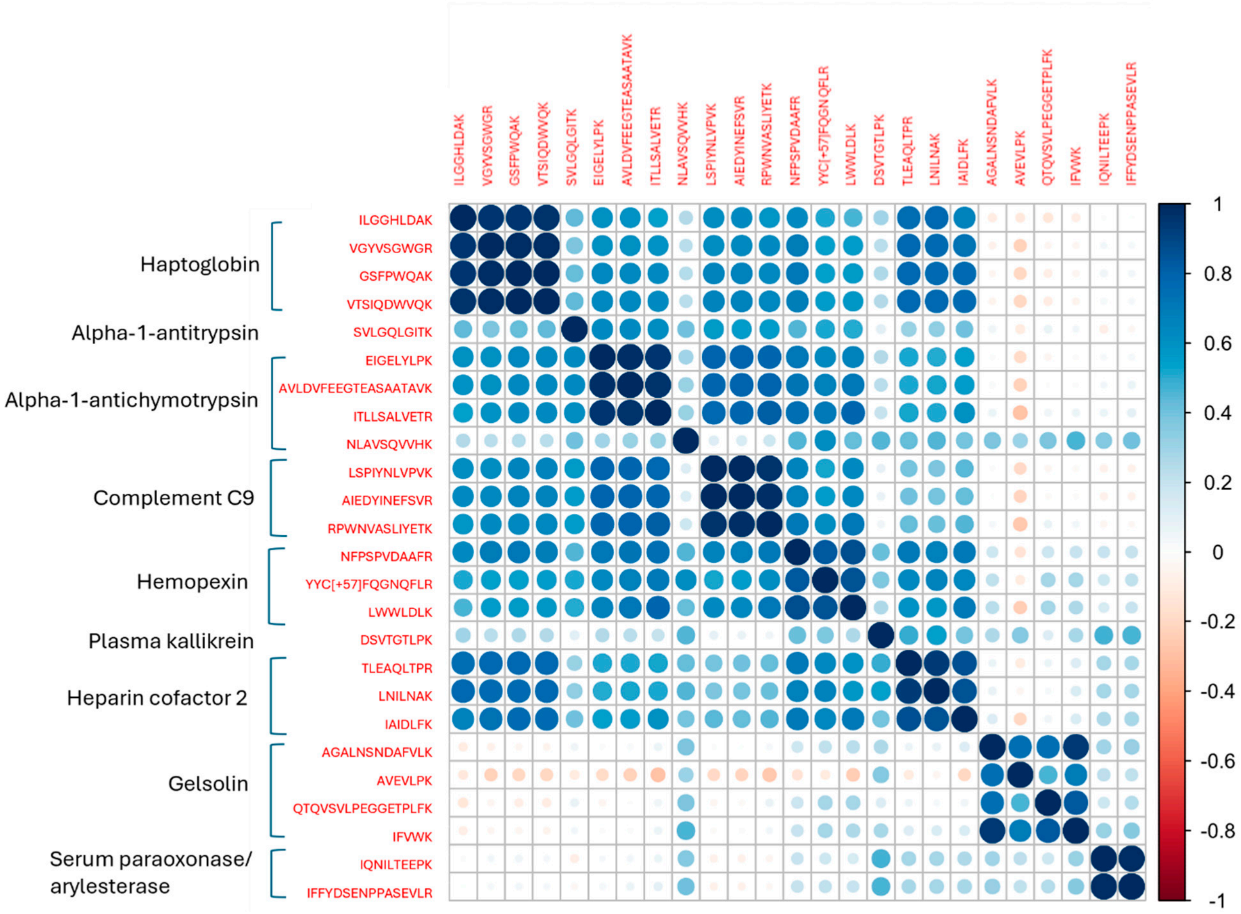

Supplementary Figure S2A-C: Model calibration: Calibration of model in development and validation cohorts. Goodness of fit assessment of calibration curves (using LOESS fit) of PromarkerEso algorithm in (A.) Development Cohort, PROBE-NET, (B.) Validation Cohort 1, Ochsner and (C.) Validation Cohort 2, VCB with 95% confidence intervals. Representative of predicted probability distributions of the model by comparing frequency of actual EAC samples with PromarkerEso predicted probability scores. Triangles represent clusters of 10 samples. Calibration in the PROBE-NET cohort was internally validated using bootstrapping to adjust for optimism-bias. EAC = esophageal adenocarcinoma, NC = control by negative endoscopy test and GenPop = general population control sample.

A. Calibration of PromarkerEso in PROBE-NET cohort (optimism-bias adjusted)

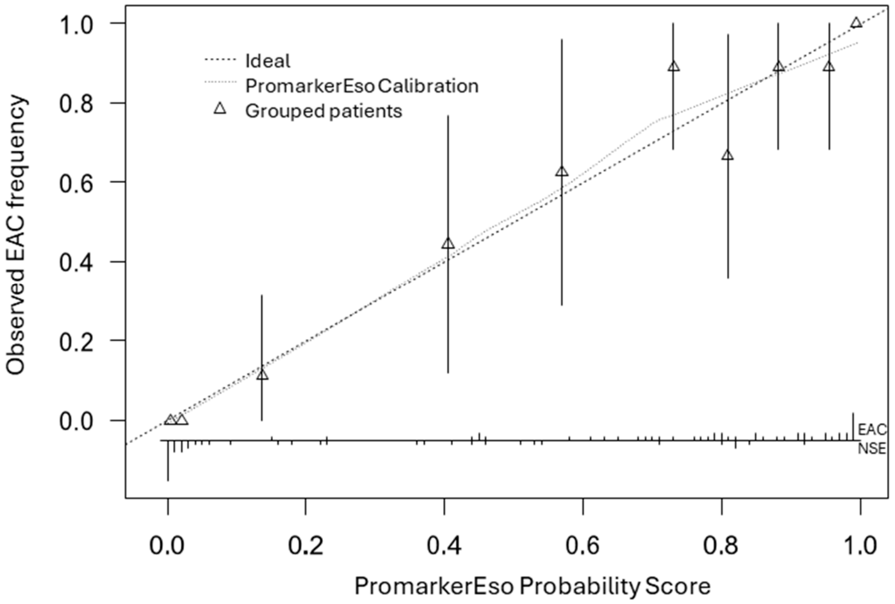

B. Calibration of PromarkerEso in Ochsner cohort

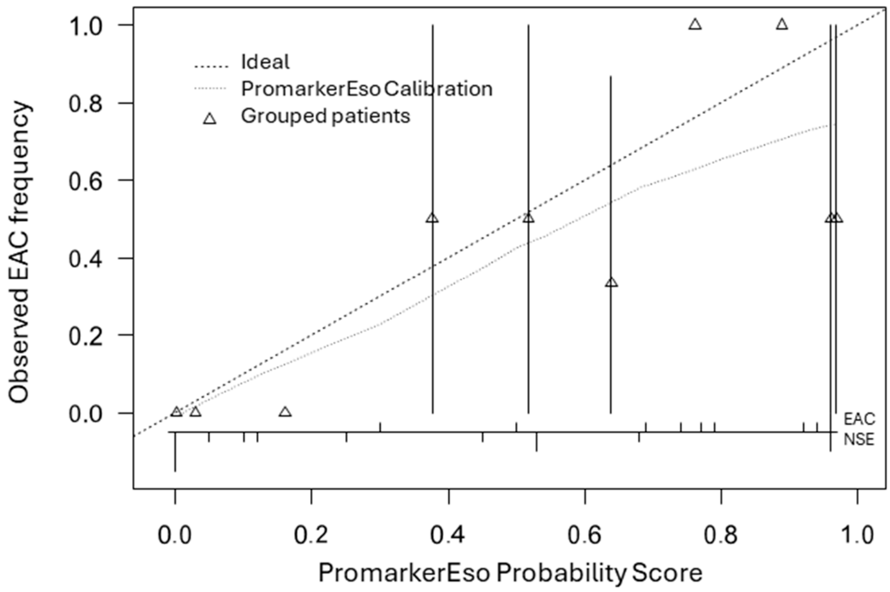

C. Calibration of PromarkerEso in VCB cohort

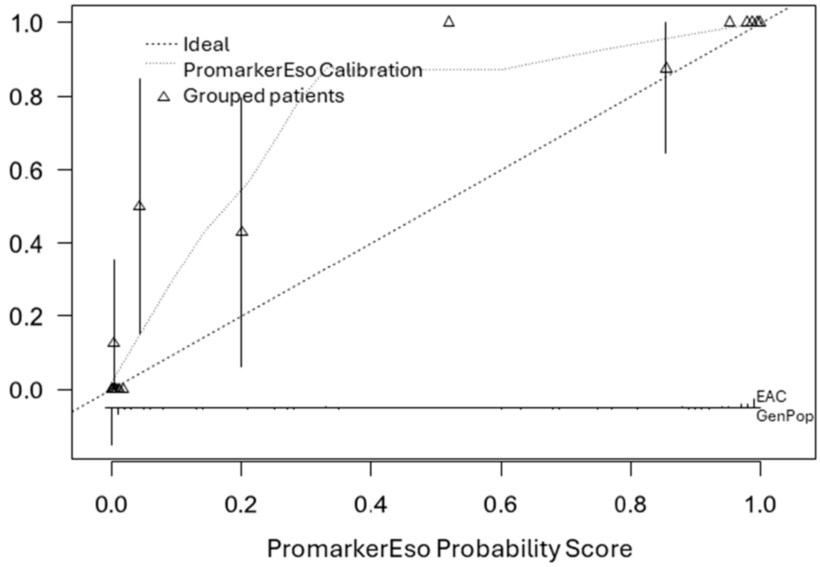

Supplement: Supplementary file 1 [file proteomes-13-00023-s001.zip › proteomes-3588418-supplementary.pdf]
